# Comprehensive Predictions of Mef2-Mediated Chromatin Loops, Which May Inhibit Ubx Binding by Blocking Low-Affinity Binding Sites

**DOI:** 10.3390/jdb12040033

**Published:** 2024-12-09

**Authors:** Katrin Domsch

**Affiliations:** Developmental Biology, Heidelberg University, COS, 69120 Heidelberg, Germany; katrin.domsch@cos.uni-heidelberg.de

**Keywords:** Mef2, Ubx, chromatin loops

## Abstract

Gene regulation depends on the interaction between chromatin-associated factors, such as transcription factors (TFs), which promote chromatin loops to ensure tight contact between enhancer and promoter regions. So far, positive interactions that lead to gene activation have been the main focus of research, but regulations related to blocking or inhibiting factor binding are also essential to maintaining a defined cellular status. To understand these interactions in greater detail, I investigated the possibility of the muscle differentiation factor Mef2 to prevent early Hox factor binding, leading to the proper timing of regulatory processes and the activation of differentiation events. My investigations relied on a collection of publicly available genome-wide binding data sets of Mef2 and Ubx (as the Hox factor), Capture-C interactions, and ATAC-seq analysis in *Mef2* mutant cells. The analysis indicated that Mef2 can form possible chromatin loops to Ubx-bound regions. These regions contain low-affinity Ubx binding sites, and the chromatin architecture is independent of Mef2’s function. High levels of Ubx may disrupt the loops and allow specific Ubx bindings to regulate defined targets. In summary, my investigations highlight that the use of many publicly available data sets enables computational approaches to make robust predictions and, for the first time, suggest a molecular function of Mef2 as a preventer of Hox binding, indicating that it may act as a timer for muscle differentiation.

## 1. Introduction

Gene regulation is established by tight communications between promoters and enhancers or cis-regulatory modules (CRMs), which are stabilised by the interactions of associated proteins [1]. These proteins can act as activators or repressors of gene transcription. To regulate gene activity, repressors can block or quench the activator’s function directly at the promoter [2,3] or in regulatory regions such as enhancers by forming chromatin loops with other elements, as has been shown for blocking insulators, including CTCF binding sites and gypsy elements in *Drosophila* [4]. This indicates that gene regulation is organised in distinct configurations regardless of the activation or inhibition of transcription. These chromatin conformations highlight the possibility that some unexplored transcription factor (TF) binding events might refer to blocking/repressive functions rather than activating functions.

During *Drosophila* development, the mesoderm is determined and specified, and the muscles differentiate through a tightly controlled network of TFs, where the activation and repression of genes are essential to form a cell lineage, stabilise it, and maintain its differentiated function. Twist (Twi), as the mesodermal master regulator, initiates a network of activation that will give myoblasts a specific fate. This fate is maintained and stabilised by Myocyte enhancer factor 2 (Mef2) and the Hox factor Ultrabithorax (Ubx) in the abdominal segments [5,6]. Both factors will promote the formation of functional muscle fibres.

Ubx protein is expressed and functionally required during myoblast specification events (stages 10–12, 4–8 h after egg laying (AEL)). Mef2, a member of the MADS-box TF family, is expressed in the mesoderm right after gastrulation (stage 9, 4 h AEL) [7]. It is activated by Twi [8], and normal mesoderm and muscle development is guided by Mef2 since *Mef2* mutant investigations indicate no muscle formation related to disrupted differentiation events (stages 14–17, 10–16 h AEL) such as myoblast fusion and muscle formation [5]. These results indicate that Mef2 might bind to chromatin regions to guide or block TFs during mesoderm specification (stages 10–12). This assumption is partially supported by detailed Mef2 chromatin binding investigations, which indicate that CRMs can be occupied early (mesoderm specification) but not late (muscle differentiation) and late but not early chromatin regions [9]. Sandmann et al. discuss early binding through the occupancy of Mef2 with Twi to activate/maintain gene activity; still, the results do not exclude a possible blocking function of Mef2, leading to the following question: what might be inhibited/blocked by Mef2?

During *Drosophila* embryonic development, it has been shown that Ubx is essential for inactivating *twi* and promoting coordinated muscle differentiation. Ubx can bind to the *twi* promoter through interactions with the NK4 homeodomain TF Tinman despite the presence of Mef2 [10]. The loss of Ubx or Tin function leads to the significant up-regulation of *twi* in differentiating muscles, whereas the loss of *Mef2* function leads to a significant reduction in *twi* expression as early as during mesoderm specification, which could be further enhanced through the overexpression of Ubx in *Mef2* mutants at stage 10, where *twi* expression is normally unaffected [10]. The results of these examples indicate that Ubx binding might be blocked by Mef2 during early specification events in an indirect fashion since Ubx and Mef2 are not directly bound to the same *twi* promoter region [10]. To investigate this hypothesis in more detail on a genome-wide scale, I collected Ubx and Mef2 genome-wide binding data, combined them with Hi-C predicted enhancer–promoter interactions, and associated them with chromatin accessibility profiles from mesodermal cells devoid of *Mef2* function.

This comprehensive analysis indicated that Mef2 might initiate enhancer–promoter loops to prevent Ubx binding. High levels of Ubx may be able to break these loops through interactions at low-affinity sites, supporting the theory that Ubx low-affinity binding is required for tissue-specific functions.

In summary, this investigation indicates that chromatin loops may also prevent Hox binding at specific time points and that high levels of Hox factors break the loops to perform spatial and temporal specific gene regulations.

## 2. Materials and Methods

### 2.1. Fly Stocks and Antibody Staining

Embryonic antibody staining was performed on the *w^1118^* fly line (BL3605). The staining protocol, in brief, was as follows: Dechorionised embryos were fixed in a fixing solution (comprising 2.8 mL of water, 400 µL of 10× PBS, 800 µL of 37% formaldehyde, and 8 mL of n-Heptane) for 20 min. Afterwards, the fixative was removed, and the vial was filled with 10 mL of Methanol. The glass vial was heavily shaken to remove the vitellin membrane; then, the embryos were washed in Methanol. For staining, the embryos were rehydrated in 50% Methanol/1× PBT (PBS with 0.1% Tween-20) and washed thrice in 1× PBT for 10 min. Next, the embryos were blocked with 1% BSA/1× PBT and incubated with the primary antibody overnight. The next day, the embryos were washed thrice in 1× PBT for 20 min and incubated with the secondary antibody for 2 h. Afterwards, the samples were washed again thrice in 1× PBT for 20 min and mounted in VectaShield. The following antibodies were used: Rb-Mef2 (1:1500, a gift from H. Nguyen, distributed by K. Domsch) and gp-Ubx (1:500, [6]). The embryos were imaged using an SP8 Leica Confocal microscope, and the images were further processed using Fiji [11].

### 2.2. Bioinformatic Analysis and Visualisation

The data used in the manuscript included Ubx ChIP-Seq and Histone ChIP-Seq NCBI Gene Expression Omnibus GSE121752 [6], enhancer–promoter interaction EMBL-EBI’s ArrayExpress E-MTAB-9310 (Capture-C data), and E-MTAB-12639 (ChIP–seq data) [12]. Mef2 ChIP-Seq ArrayExpress: E-TABM-57. Sci-ATAC ArrayExpress: E-MTAB-9034 [13]. All data were annotated against genome dm6. The data sets contained mesodermal Ubx ChIP-Seq peaks at stages 10–13, Mef2 binding peaks combined with stages 10–12, mesodermal histone mark disruptions of H3K27ac at stages 10–13, and single-cell ATAC-Seq data of Mef2 mutants in a pseudo-bulk. Bioinformatics analysis was performed as described in [6]. In general, the following tools were used for this analysis: SAMtools [14], BEDtools (intersect, window, and coverage [15]), motif search by the MEME suite web tool (Frith et al., 2008), and deepTools [16]. It was visualised with IGV, PANTHER (GO biological function complete, Binomial), Fisher, FDR correction [17,18,19,20,21], and the WEADE tool for higher-order GO-term enrichment [22]. R tools were also used, namely ChIPseeker [23], ChIPpeakAnno [24], and NLRB [25]. R was used to generate plots and perform Wilcox significance tests [26]. Promoters and enhancers were defined in relation to [27,28]. The online tool BioVenn [29] was also used. Single tools were also used in detail in [6,10,30]. In more detail, the accepted peaks for each data set (Ubx and Mef2) were compared/overlapped with ChIPpeakAnno to investigate the direct overlap of peaks. For an overlap of the associated genes, the peaks were associated with genes using ChIPseeker, and the resulting genes were overlapped with BioVenn to generate a Venn diagram and generate an overlapping data set. The location of the peaks with respect to the gene was investigated using ChIPseeker. The direct overlap of Ubx/Mef2 peaks and confidence sites from the Capture-C data was achieved using BEDtools intersect, identifying a direct overlap of genomic regions. The identification of regions in the distance was performed using the BEDtools window and defined kb as distance. Regions that appeared and the following distances were removed to identify the new regions for each distance. The genomic view of the twist locus, as an example, was achieved in IGV using files generated with deepTools and bed files (accepted peaks: MASCS2). GO-term analysis was performed on selected files in WEADE (using heatmap data from WEADE and illustrated in R). The same files were used for detailed GO-term analysis in PANTER (selecting for molecular or biological terms). The information for the summit was collected from the bed files, including the accepted peaks, and illustrated in R. The NRLB algorithm was adapted to investigate more reads simultaneously and identified 10 low-affinity peaks per genomic region. The data were collected and illustrated in R. Wilcox’s test was performed in R. The investigations of the peak coverage were performed on selected data sets using BEDtools coverage. Then, the data were collected and illustrated in R. The motif analysis used different data sets, which were uploaded into the web tool MEME suite, and motifs were selected and illustrated.

## 3. Results

### 3.1. Ubx and Mef2 Binding Peaks Rarely Overlap but Regulate Similar Genes

**Figure 1:**
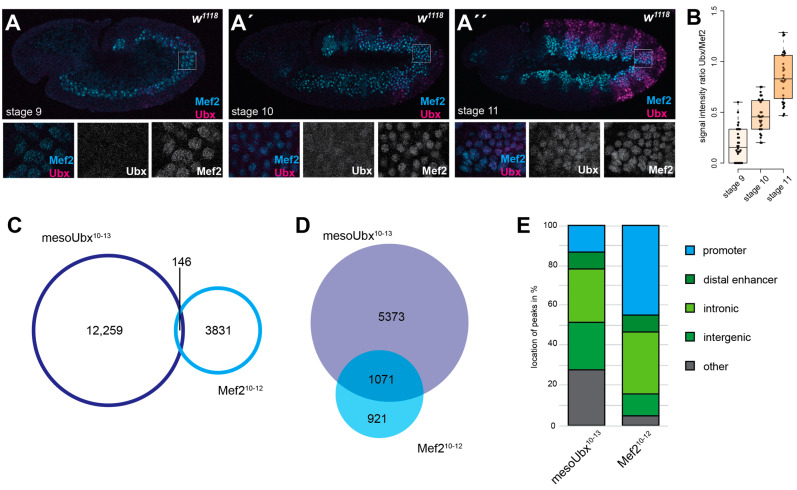
Ubx and Mef2 bind to distinct regions but could regulate common genes. (**A**) Embryonic staining: Ubx, Mef2, (**D**) stage 9, (**A′**) stage 10, (**A″**) stage 11, small, high-resolution images (square). (**B**) Box plot: signal intensity ratio of Ubx/Mef2 at different stages. (**C**) Venn diagram: genetic regions associated with Ubx and Mef2 binding. (**D**) Venn diagram: overlap of genes associated with Ubx and Mef2 binding. (**E**) Bar diagram: mesodermal Ubx and Mef2 peak localisation focus on gene body. (Classification: promoters: −1000 to +10 bp from TSS and 5′ UTR; distal enhancers: −2000 to −1000 bp from TSS, 3′ UTR, and downstream; intron: intronic regions; intergenic: distal intergenic; other: including exons).

Ubx and Mef2 protein expression was detected throughout embryonic development after their initiation, which was around stage 9 for Mef2 and stage 10/11 for mesodermal Ubx. Both proteins were colocalised during mesoderm specification events (Figure 1A″, stage 11) and were present in a substantial amount (Figure 1B). Further, no Ubx expression was detectable in Mef2-positive nuclei at stage 9, and very low Ubx levels were noted at stage 10 (Figure 1A,A′). The ratio between Ubx and Mef2 indicated an increase in Ubx expression over the developmental stages in Mef2-positive nuclei (Figure 1B), highlighting that the initiation of the Mef2 expression was independent of Ubx. These results proposed the possibility that Mef2 might bind earlier to Ubx-associated regions to block them and to prevent Ubx occupation. To further investigate this assumption, I reanalysed mesodermal Ubx ChIP-Seq [6] and Mef2 ChIP-on-ChIP data [9,13]. An overlap of both binding peaks indicated that these peaks were very distinct from each other, with very few common binding events (Figure 1C). The analysis of the peak-associated genes indicated that a substantial number of genes were commonly bound (Figure 1D).

An overall investigation of binding peak locations also highlighted the differences between the two transcription factors. Ubx mainly bound at enhancer regions (distal enhancers, intronic, and intergenic), and Mef2 peaks were found to be associated with promoters and enhancers in an equal proportion (Figure 1E). These observations indicated that it was very unlikely that Mef2 would directly bind/block Ubx binding regions. The substantial number of common genes indicated that other chromatin structures, such as loops, might be used by Mef2 to prevent low amounts of Ubx protein from binding these genomic regions.

### 3.2. Chromatin Loops Mediated by Mef2 Prevent Ubx Binding Gain Tissue Specificity over Distance

**Figure 2:**
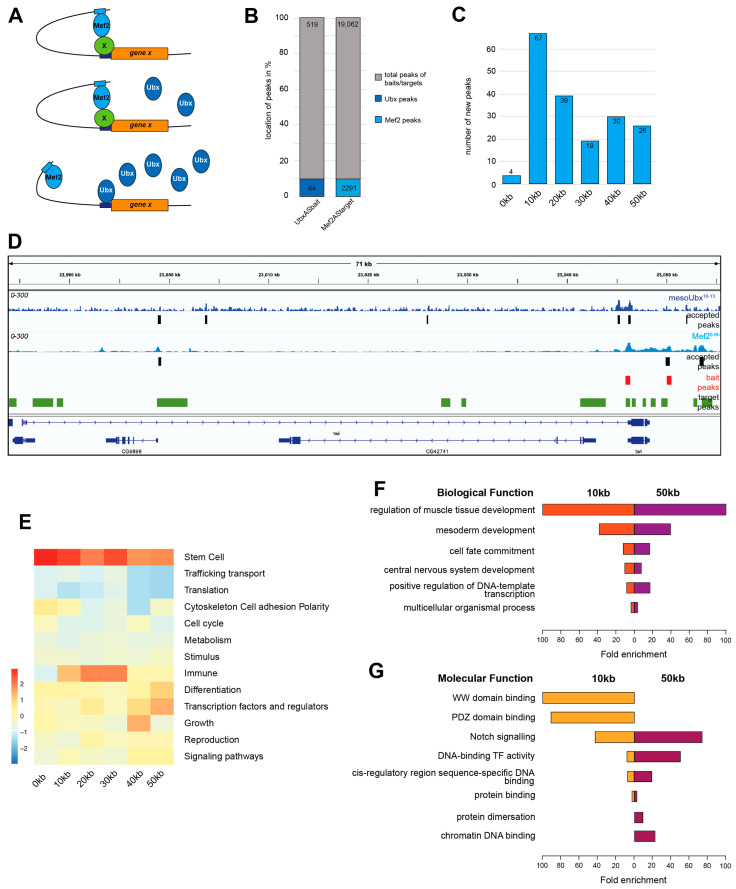
Association of Ubx and Mef2 binding to enhancer–promoter loops. (**A**) Theoretical idea of Ubx-Mef2 enhancer loops. Mef2 prevents Ubx binding, with high Ubx concentrations breaking the loop. (**B**) Bar diagram: Ubx and Mef2 binding associated with known enhancer–promoter loops. Ubx is the bait (UbxASbait), and Mef2 is the target (Mef2AStarget). (**C**) Bar diagram: Ubx and Mef2 enhancer–promoter interactions at 10 kb, 20 kb, 30 kb, 40 kb, and 50 kb distance. (**D**) Gene view: twist gene locus showing Ubx (dark blue) and Mef2 (light blue) binding peaks and accepted bait (red) and target (green) regions. (**E**) Heat map: higher-order GO-term clustering between different enhancer–promoter interactions according to the distance. (**F**,**G**) Bar plots: detailed GO-term analysis with respect to biological terms (**F**) and molecular terms (**G**) comparing 10 kb and 50 kb distances.

Ubx and Mef2 chromatin binding peaks did not overlap, and to investigate the assumption that Mef2 might prevent Ubx binding, I used the concept that enhancers interact with promoters by forming chromatin loops [31]. The idea was that Mef2-bound enhancer regions would interact with promoters, which could be bound by tissue-specific factors to prevent Ubx binding at low levels. The loop will be broken/released through higher dosages of Ubx protein, which will replace the tissue-specific factor and interfere with the Mef2 interaction (Figure 2A). To computationally test this hypothesis, I used the enhancer–promoter interaction data set published by Pollex et al. [12]. The data were generated by investigating genome-wide Capture-C interactions on about 600 selected enhancer–promoters (baits) that interacted with over 20,000 distal enhancers (targets) [12]. For example, the data indicate that in the *twi* genomic regions, 2 baits interact with 14 potential distal enhancers (targets/green) (Figure 2D). One of the baits (red, Figure 2D) correlated with a Ubx binding site, and two targets (green, Figure 2D) overlap with Mef2, indicating that Mef2 might form loops, which could be broken by Ubx. This idea was also supported by previously performed ChIP-qPCR experiments on the *twi* gene locus, which showed that Mef2 did not directly bind the *twi* promoter but a distal enhancer and that this binding was removed by the increased dosage of Ubx protein [10].

To link Ubx and Mef2 binding to known chromatin loops and investigate their biological requirements, I overlapped Ubx binding peaks with the selected 600 known baits and Mef2 binding peaks with the over 20,000 identified targets. About 10% of the baits and targets corresponded to Ubx or Mef2 binding events, leading to 64 Ubx binding and 2291 Mef2 binding regions for further analysis (Figure 2B). Next, I wanted to know what distance to the baits at which the targets can be located. By using BEDtools (window), which allows me to grab regions in a defined distance, I could identify and select new interactions. Most of the interactions were closer to the first 10 kb, but long-distance interactions were also possible with up to 50 kb (Figure 2C). Further, I was interested in whether these interactions had any biological relevance; for this, I associated the Ubx bait regions of the different distances with genes and clustered them in higher gene ontology (GO) terms (Figure 2E). The analysis indicated that all of the category’s genes were included with functions associated with stem cells. Genes that were controlled by 10 kb to 30 kb distance loops were also involved in the immune response, whereas genes regulated by larger distance loops, 40 kb and 50 kb, were associated with functions of differentiation and TF/regulator activity and growth, indicating that the distance of Mef2-bound regions and the loop length might be associated with the regulation of different sets of genes, which were required for defined biological functions. This assumption was supported by a closed investigation of the biological and molecular GO terms comparing 10 kb and 50 kb distances since they were remarkably different. The analysis indicated that the regulated genes were associated with very similar biological but different molecular functions. 10 kb loops regulate more genes performing protein–protein interaction (WW domain binding, PDZ domain binding) as 50 kb genes were associated with transcription factor activity and DNA/chromatin binding (Figure 2E,F).

Taken together, these investigations indicated that Mef2-bound target regions might form chromatin loops with baits, which were bound by Ubx. The interaction was possible over long distances and had biological and molecular relevance.

### 3.3. Disruption of Chromatin Loops Depends on Ubx Low Binding Affinity

**Figure 3:**
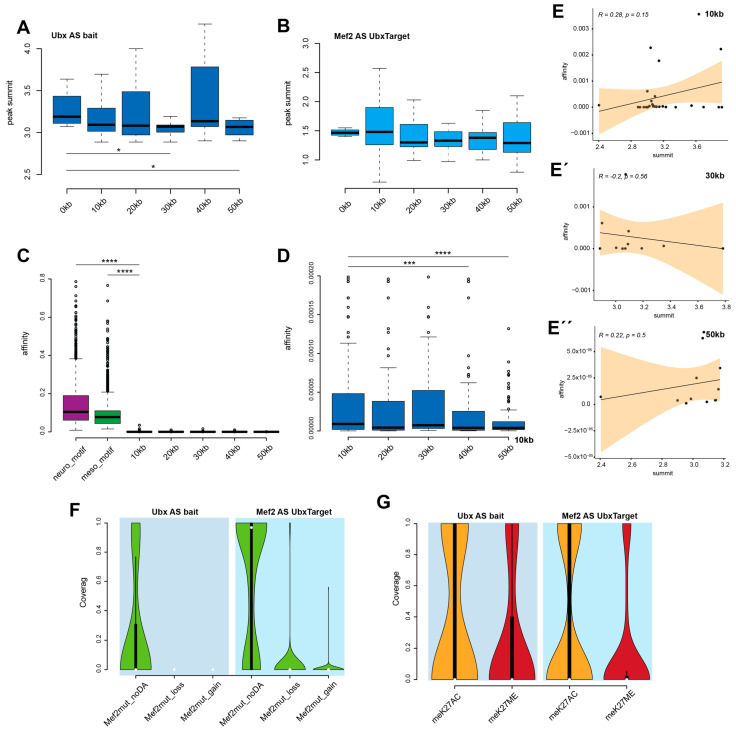
Mef2-linked enhancer–promoter interactions block low-affinity Ubx binding sites. (**A**) Box plot: Ubx peak summit distribution showing a high variability across distances (0 kb to 30 kb * *p*-value: 0.041; 0 kb to 50 kb * *p*-value: 0.030). (**B**) Box plot: Mef2 peak summit distribution showing high similarity across the distances. (**C**,**D**) Box plot: Ubx affinities analysed with the NRLB algorithm. (**C**) Box plot: Ubx bait peaks of very low affinity as compared to neuronal and mesoderm motifs identified (meso to 10 kb **** *p*-value: < 2.2 × 10^−16^, neuro to 10 kb **** *p*-value: < 2.2 × 10^−16^) (**D**) Box plot: Ubx affinity zooming into the Ubx bait regions (10 kb to 40 kb *** *p*-value: 0.0040, 10 kb to 50 kb **** *p*-value: 0.00063). (**E**) Correlation analysis: summit to affinity, 10 kb (**E**), 30 kb (**E′**), and 50 kb (**E**″). (**F**) Violin plot: peak coverage between the Ubx bait and Mef2 target peaks with Mef2 mutant ATAC data sets. (**G**) Violin plot: peak coverage between Ubx bait and Mef2 target peaks with H3K27ac and H3K27me3 ChIP-Seq data sets.

Since the formation of chromatin loops between Ubx and Mef2-bound regions were possible, I posed the following question: Did the formation/disruption depend on specific factors or even chromatin conditions? For the investigations, I analysed the peaks in more detail, taking the summit into account. The peak summit describes the highest fragment pileup and was predicted to indicate the binding location [32]. Ubx peak summits varied over distance and decreased with longer loops, indicating that these regions did not show such a large fragment amount and were less bound (Figure 3A). Mef2 peak summits were, in general, lower than the one from Ubx and kept their overall size, indicating that these regions were bound in equal strength (Figure 3B). Since the Ubx peaks demonstrated weaker binding with distance, I wondered if this correlated with their binding affinities. It has been shown for Ubx that low protein binding affinity was associated with high specificity, meaning that a specific function is often associated with low-affinity binding [33]. To estimate the affinities of the Ubx-bound regions, I used the No Reads Left Behind (NRLB) algorithm and compared the affinities with previously identified Ubx motifs in the neuronal (neuro_motif) and mesodermal (meso_motif) tissues with the newly investigated regions (Figure 3C) [30]. The results indicated that the Ubx bait regions were of significantly very low affinity and that the affinity was significantly decreasing with distance (Figure 3D). With respect to the reduced summit at higher distances, the reduction in affinity was moderately correlated (Figure 3E,E′,E″), indicating that with larger loops, both binding amounts and affinities decreased.

Genome interactions and transcription regulations depend on the overall chromatin environment and architecture. The ATAC-seq (Assay for Transposase-Accessible Chromatin using sequencing) method and the resulting data can provide insight into chromatin accessibilities. Since Mef2 was expressed earlier than Ubx in the mesoderm (Figure 1A), I wanted to investigate if Ubx binding and the accessibility of the chromatin depended on Mef2 function. To this end, I used the available scATAC-Seq generated from *Mef2* mutant embryonic mesodermal cells [13] by comparing the coverage of the *Mef2* mutant ATAC pseudo-bulk regions with the Ubx bait regions and Mef2-bound targets (Figure 3F). In addition, I clustered the *Mef2* mutant ATAC data in (1) regions with no changes (Mef2mut_noDA), (2) regions that lose accessibility (Mef2mut_loss), and (3) regions that gain accessibility (Mef2mut_gain) (Figure 3F). The results showed that the chromatin accessibility of the Ubx baits did not change upon losing the *Mef2* function and that most of the Mef2-bound regions remained at their chromatin status regardless of the *Mef2* function. A small proportion of Mef2-bound targets lost or gained accessibility (Figure 3F). Since there were no changes in the chromatin environment with respect to the *Mef2* function on the Ubx bait or Mef2 target regions, I questioned the general status of these baits and targets with respect to their histone modifications, which can be linked to open (H3K27ac) and/or closed/repressed (H3K27me3) chromatin regions. For these investigations, I analysed the coverage between Ubx baits, Mef2 targets, and histone peaks (H3K27ac: active (meK27AC), H3K27me3: inactive/repressed (meK27ME)) by using mesodermal-specific histone-ChIP-Seq data [6]. The results indicated that most of the bait and target regions were open and accessible; only a small fraction of Ubx-bound regions were closed and covered with H3K27me3 marks (Figure 3G).

In sum, the results highlighted that Ubx binding to defined bait regions depended on low-affinity binding sites. These regions were open and accessible, and the binding ability did not depend on the *Mef2* function. Further, Mef2-bound targets were open regions, and these chromatin environments were mostly independent of Mef2 protein binding abilities.

## 4. Discussion

**Figure 4:**
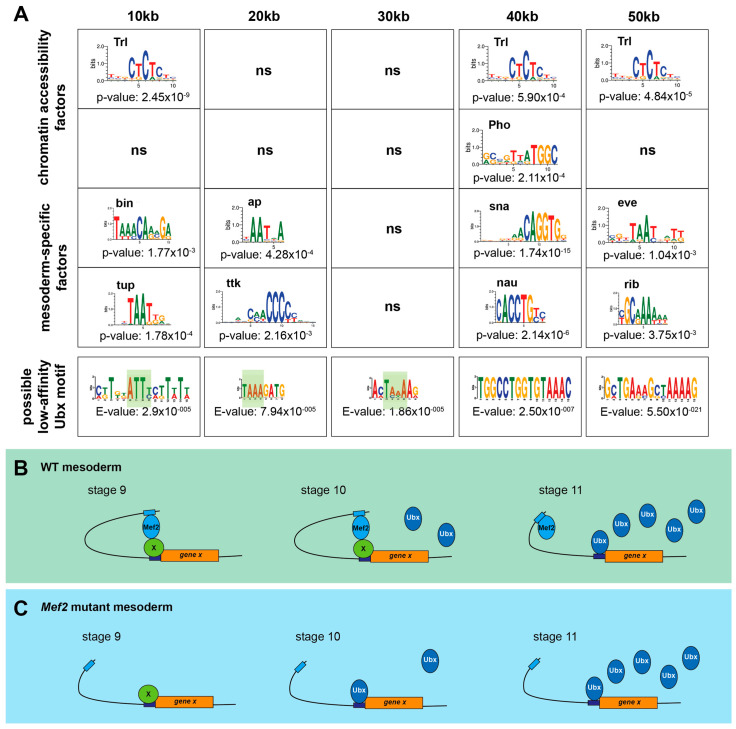
The breaking of Mef2-linked enhancer–promoter interactions by Ubx may depend on different mesodermal-specific transcription factors. (**A**) Motif search (MEME): specific mesodermal factors for each distance, known chromatin accessibility factors. In addition, de novo motif search on potential low-affinity Ubx binding site motifs. (**B**,**C**) Working model: opening of the Ubx and Mef2-linked enhancer–promoter interactions. (**B**) wildtype situation in three different stages. Stage 9: no Ubx, Mef2 can mediate the interaction to specific transcription factors. Stage 10: low Ubx concentration; it cannot bind to the region. Stage 11: high Ubx concentration can open/break the loop and regulate the following gene. (**C**) Situation in the Mef2 mutant according to the ATAC data. Stage 10: Ubx can bind to the region with low amounts and regulate associated genes. ns: non-significant.

In this study, I showed that two transcription factors, which were co-expressed in the mesodermal tissue, have very little in common with respect to their binding architecture. Of course, Ubx, as a homeodomain TF, binds to a selected set of sites, and Mef2, a MADS-box family member, binds to totally different regions, but it is known that Hox factors achieve their specificity through interactions with co-factors and collaborators [34]. Since Mef2 is expressed throughout mesoderm and muscle development and is an essential function for muscle differentiation, I assumed that Mef2 could be a mesodermal co-factor or collaborator for Ubx. This hypothesis largely focuses on a positive interaction and denies a negative relationship between Ubx and Mef2. This negative interaction became clear through investigations of *Mef2* mutants with respect to Ubx-mediated *twi* repression [10]. In *Mef2* mutants, stronger and earlier repression of *twi* was detected, highlighting the possibility that early Ubx binding is prevented/blocked by Mef2. Since the two factors are not co-binding, Mef2 might initiate the formation of chromatin loops with Ubx-bound regions to prevent Ubx binding at low levels (Figure 4B). This interaction would, for example, prevent the early repression of *twi* at stage 10 to inhibit the early activation of muscle differentiation events [10]. The tissue-specific factor Mef2 used in this example is Tinman, because Tinman and Ubx genetically interact to initiate the repression of twi [10].

Mef2 could interact with new regions on the basis of different mesodermal factors, which reflect a relationship between loop length and possible genes associated with biological and molecular functions (Figure 2 and Figure 4A). Based on my analysis, Mef2 might interact with forkhead TF binou (bin), Zink finger TFs (tailup (tup), apterous (ap), tramtrack (ttk), snail (sna)), homeodomain TFs (tailup (tup), apterous (ap), even skipped (eve)), BTB/POZ domain TFs (tramtrack (ttk), ribbon (rib)), and the basic Helix-loop-Helix domain TF nautilus (nau). The interaction between Mef2 and bHLH transcription factors has been extensively studied due to the connections of both protein families to skeletal muscle development, supporting my results [35]. Since some of these proteins belong to the homeodomain TF family and bind to very similar regions, it is possible that these factors block Ubx binding at low amounts. High levels of Ubx can compete and break the chromatin loop. In line with these assumptions is the identification of Ubx low-affinity binding motifs, which are a variation in the ATTA-core/classical motif (Figure 4A), highlighting the possibility for other homeodomain TFs to bind these regions and compete with Ubx. In *Mef2* mutants, Ubx might have a higher possibility of binding to these regions with low protein levels, thereby allowing it to perform its function (Figure 4B), indicating a possible molecular function for Mef2 in the context of Ubx as a preventer/inhibitor of Ubx binding.

Early Mef2 binding (4–6 h AEL) might not be required for general Mef2 function but may be essential for embryonic development [9]. Sandmann et al., 2006, postulated that Mef2 occupies Twi binding sites and that the interaction between these two might be important for target gene regulation. The authors were able to identify 42% of the Mef2 binding sites close to Twist, leaving 58% of early Mef2 binding unrelated to Twi [9]. Since I could not identify Twi binding motifs enriched in my investigations, it is possible that the 58% of Mef2 binding sites function as TF binding inhibitors in a chromatin loop fashion or through direct chromatin interactions. I could only demonstrate a very small proportion, although the restriction to Ubx as bait shows that chromatin interactions and blocking are possible.

Further investigations into the biological relevance of Mef2-mediated chromatin loops and direct proof that these structures are built are required. 3C experiments on the regions identified in this study might yield a direct correlation between the formation and the biological relevance of these chromatin organisations [36]. In addition, DNA FISH coupled with fluorescence detection could be used to show the interactions of single regions with each other [37].

Overall, my investigations suggest that Mef2 may function as a timer for TF binding by preventing Ubx chromatin interactions through the formation of loops and the interaction of additional tissue-specific factors. This possibility provides developmental and biological relevance for early Mef2 binding events.

## Data Availability

The data presented in this study are available in: Ubx ChIP-Seq and Histone ChIP-Seq NCBI Gene Expression Omnibus GSE121752, Enhancer-Promoter interaction EMBL-EBI’s ArrayExpress E-MTAB-9310 (Capture-C data) and E-MTAB-12639 (ChIP–seq data). Mef2 ChIP-Seq ArrayExpress: E-TABM-57 and sci-ATAC ArrayExpress: E-MTAB-9034. All data was annotated against genome dm6.
